# Microvesicle removal of anticancer drugs contributes to drug resistance in human pancreatic cancer cells

**DOI:** 10.18632/oncotarget.10395

**Published:** 2016-07-04

**Authors:** Vandhana Muralidharan-Chari, Hamed Gilzad Kohan, Alexandros G. Asimakopoulos, Thangirala Sudha, Stewart Sell, Kurunthachalam Kannan, Mehdi Boroujerdi, Paul J. Davis, Shaker A. Mousa

**Affiliations:** ^1^ The Pharmaceutical Research Institute, Albany College of Pharmacy and Health Sciences, Rensselaer, NY 12144, USA; ^2^ Department of Pharmaceutical Sciences, Albany College of Pharmacy and Health Sciences, Albany, NY 12208, USA; ^3^ Wadsworth Center, New York State Department of Health, and School of Public Health, University at Albany, Albany, NY 12201, USA; ^4^ Department of Medicine, Albany Medical College, Albany, NY 12208, USA

**Keywords:** drug expulsion, drug resistance, microvesicles, pancreatic cancer, transporter proteins

## Abstract

High mortality in pancreatic cancer patients is partly due to resistance to chemotherapy. We describe that human pancreatic cancer cells acquire drug resistance by a novel mechanism in which they expel and remove chemotherapeutic drugs from the microenvironment via microvesicles (MVs). Using human pancreatic cancer cells that exhibit varied sensitivity to gemcitabine (GEM), we show that GEM exposure triggers the cancer cells to release MVs in an amount that correlates with that cell line's sensitivity to GEM. The importance of MV-release in gaining drug resistance in GEM-resistant pancreatic cancer cells was confirmed when the inhibition of MV-release sensitized the cells to GEM treatment, both *in vitro* and *in vivo*. Mechanistically, MVs remove drugs that are internalized into the cells and that are in the microenvironment. The differences between the drug-resistant and drug-sensitive pancreatic cancer cell lines tested here are explained based on the variable content of influx/efflux proteins present on MVs, which directly dictates the ability of MVs either to trap GEM or to allow GEM to flow back to the microenvironment.

## INTRODUCTION

Microvesicles are a class of extracellular vesicles (EVs) with a phospholipid membrane bilayer and are released by all types of cells. There is not yet a general, accepted classification of EVs because of a lack of specific markers and characteristics that can define the subsets of EVs, namely, microparticles, microvesicles, exosomes, oncosomes, and apoptotic bodies. Although there are challenges in the identification and characterization of EVs, the release of EVs by cells occurs in a regulated manner [1, 2]. Based on the method of isolation and morphology [3–7], the class of EVs in this study will be referred to as microvesicles (MVs). MVs are derived from external budding and pinching of plasma membrane and contain specific antigens that are unique to their cells' origin [8]. MVs play a significant role in both tumor survival and progression [1, 9–14].

MVs enable acquisition of drug resistance in cancer cells by transferring drug transporter proteins MRP1 and P-glycoprotein (P-gp) from drug-resistant cancer cells to drug-sensitive cancer cells [15–17]. Release of exosomes was shown to be a mechanism in acidosis-mediated cisplatin resistance in human melanoma cells [18, 19]. In colon cancer cells, enhanced secretion of miR-145 and miR-34a via MVs increased the cells' resistance to 5-fluorouracil [20]. In an attempt to make vehicles to deliver anticancer agents, exosomes released by mesenchymal stem cells treated with paclitaxel were found to contain paclitaxel [21]. These findings suggested a role for MVs in cellular drug resistance, but the actual mechanism is not understood.

Here, we identify that MVs facilitate the removal of therapeutic drugs both from human pancreatic cancer cells and from their microenvironment and enable the cells to resist gemcitabine (GEM). Using a panel of human pancreatic cancer cells with graded ability to resist GEM, we show that (i) GEM triggers the release of MVs in an amount that correlates with the cells' ability to resist GEM, (ii) MV-release is essential to GEM resistance because inhibition of MV-release in drug-resistant pancreatic cancer cell lines sensitizes cells to GEM both *in vitro* and *in vivo*, (iii) GEM regulates the amount of both influx and efflux proteins on MVs, and (iv) MVs remove drugs that enter cells and drugs present in the microenvironment. Based on the composition of influx and efflux proteins on MVs, the ability of MVs to trap the drugs (when released by GEM-resistant cells) or to allow the drugs to flow back to the microenvironment (when released by GEM-sensitive cells) was differentiated among the studied pancreatic cancer cell lines.

## RESULTS

### Pancreatic cancer cells release MVs in an amount that correlates with their ability to resist GEM

**Figure 1:**
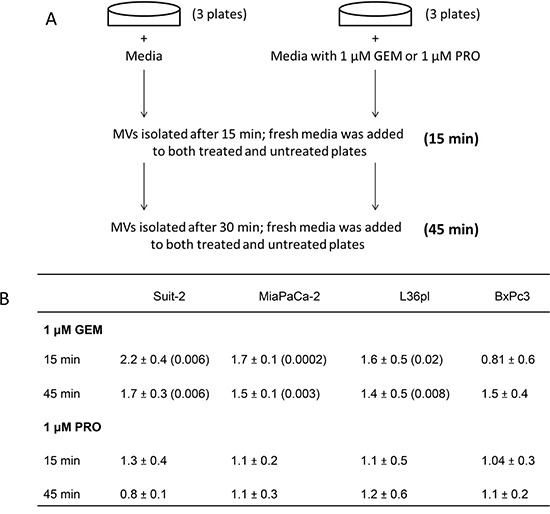
GEM treatment triggers pancreatic cancer cells to release MVs, which correlates to the cells' ability to resist GEM **A.** Illustration of the experiment. **B.** After treatment, MVs were isolated from the cells after 15 min and 45 min and were quantitated based on their surface proteins. P values are compared with their respective untreated group. Results are presented as mean ± SD. GEM = gemcitabine; PRO = procainamide.

The degree of GEM resistance by six pancreatic cell lines was determined after treating them with 1 μM GEM and calculating their percentage of cell death. We chose GEM because it is often used as first-line therapy, though it is palliative in patients with metastatic pancreatic cancer. GEM resistance was MPanc-96 = Suit-2 = Capan-2 > MiaPaCa-2 > L36pl > BxPc3 (Supplemental Figure S1). We then tested if there was any correlation between the degree of drug resistance and the cells' ability to release MVs, as outlined in Figure 1A. Suit-2, MiaPaCa-2, L36pl, and BxPc3 were used because they represent a continuum of resistance to GEM. Cells were treated with either GEM or procainamide, an anti-arrhythmic drug [22]. In response to treatment with GEM for 15 min, the amount of MVs released by Suit-2, MiaPaCa-2, and L36pl cells was significantly higher compared to the amount of MVs released by untreated control or procainamide-treated cells. There was no significant increase in the release of MVs by BxPc3 cells after 15 min. After 45 min, the amount of MVs released by Suit-2 and MiaPaCa-2 cells was significantly higher compared to the amount of MVs released by procainamide-treated cells. There was no significant increase in the release of MVs by L36pl and BxPc3 cells after 45 min (Figure 1B). Collectively, the amount of MVs released by pancreatic cancer cells in response to GEM correlates to the cells' ability to resist GEM; the greater the resistance, the larger the amount of MVs released. No such correlation was seen in response to procainamide.

**Figure 2:**
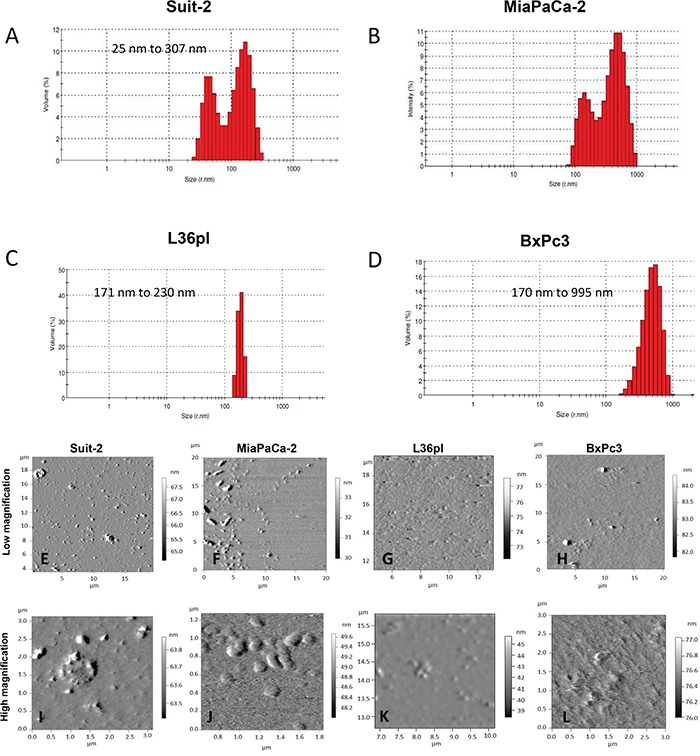
Pancreatic cancer cells release MVs Dynamic light scattering analysis of MVs isolated from **A.** Suit-2 cells **B.** MiaPaCa-2 cells **C.** L36pl cells **D.** BxPc3 cells. The AFM images of isolated MVs from each cell line are shown at low magnification **E-H.** and high magnification **I-L.** Note the presence of distinct heterogeneity in MVs at low magnification.

To characterize the isolated MVs, we determined their size distribution using dynamic light scattering (DLS). MVs released by cells ranged from 23 – 995 nm (Figure 2A-2D). Because the sizes obtained with DLS cannot distinguish membrane vesicles from co-isolated non-membranous particles of similar size, we used atomic force microscopy (AFM) both to confirm the presence of membrane vesicles and to provide an indication of the heterogeneity and morphology of the vesicle preparation. Wide field AFM images of MVs (Figure 2E-2H) reveal the heterogeneity of the vesicles and confirm the data obtained from DLS analysis. Close-up images of MVs (Figure 2I-2L) show single membranous vesicles and clustered vesicles, revealing the morphology and membranous nature of the vesicles isolated.

### Therapeutic drugs are detected in released MVs

**Figure 3:**
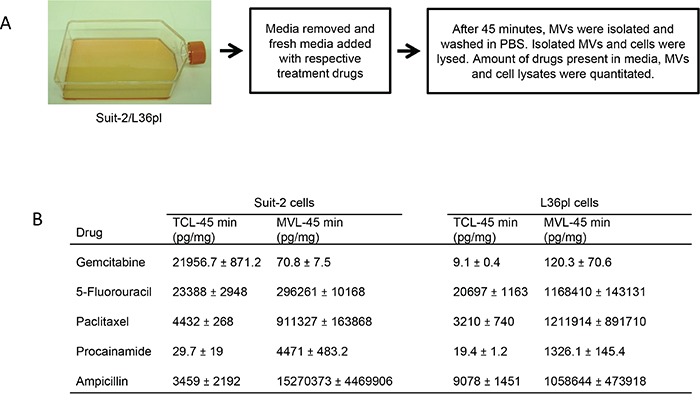
Microvesicles contain therapeutic drugs **A.** Experimental illustration for isolation of MVs released by Suit-2 and L36pl cells. **B.** The amount of drugs present in the media, in total cells, and in released MVs was estimated with HPLC-MS/MS and is expressed as pg/mg of total proteins. Results are presented as mean ± SD. Experiments were repeated twice, in duplicate. Estimation of drugs in ng/mL is in Supplemental Fig. S2. TCL = total cell lysates; MVL = MV-lysates.

To test the hypothesis that pancreatic cancer cells use MVs to remove drugs to mitigate the intracellular concentration, we treated Suit-2 (most resistant to GEM) and L36pl cells (least resistant to GEM) with GEM for 45 min and analyzed for GEM in the released MVs. GEM was present in the MVs released by both Suit-2 and L36pl (Figure 3A). To confirm that this phenomenon is not unique to GEM, we used 5-fluorouracil and paclitaxel, which are routinely used in the treatment of pancreatic cancer. We also used procainamide, which is not specific to pancreatic cancer cells, and ampicillin, which is not specific to mammalian cells. HPLC-MS/MS analysis revealed that all the drugs were present in both the total cell lysates and in MV-lysates of Suit-2 and L36pl cells (Figure 3B & Supplemental Figure S2). Analytical details for each drug are shown in Supplemental Figures S3-S8. To prevent contamination from drugs from the outer surfaces of MVs, we rinsed MVs additionally with PBS before lysis, and the PBS wash was analyzed for drugs. Consistently, the amount of drug in the wash was 5- to 80-fold less compared to the amount detected in the corresponding lysates (data not shown). We further tested if MVs released by other cancer cell types also facilitate removal of therapeutic drugs. For this, we used FMMC 419II breast cancer cells, which are highly resistant to therapeutic drugs due to their stem cell properties [23]. Both the treated cells and the MVs they released contained doxorubicin (Supplemental Figure S9), suggesting that MVs released by breast cancer cells, in addition to pancreatic cancer cells, also facilitate removal of therapeutic drugs.

### Inhibition of MV-release sensitizes GEM-resistant pancreatic cells to GEM *in vitro*

**Figure 4:**
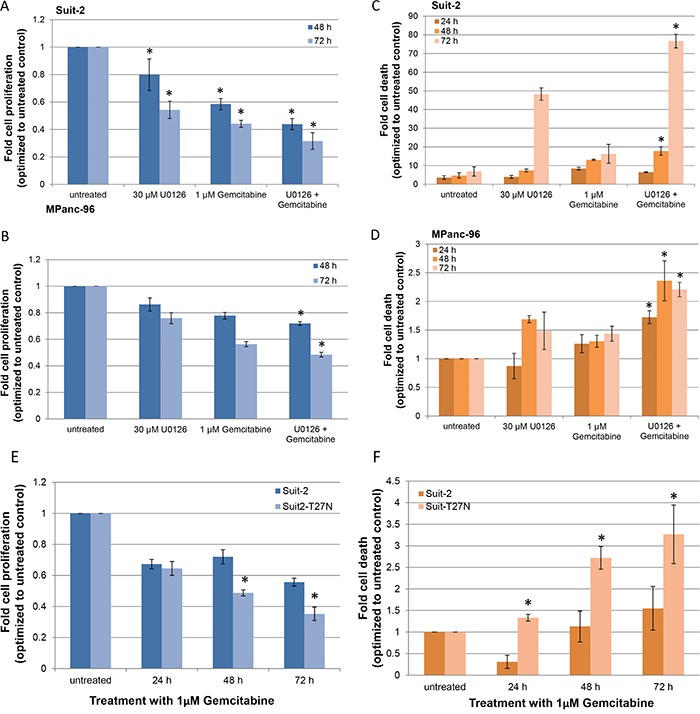
Inhibition of MV-release sensitizes drug-resistant pancreatic cancer cells to GEM *in vitro* **A.** Suit-2 cells and **B.** MPanc-96 cells were treated with either 30 μM U0126 or 1 μM GEM or both. Cell proliferation was determined after 48 h and 72 h. **C.** Suit-2 cells and **D.** MPanc-96 cells were treated as before and analyzed for cell death after 24, 48, and 72 h by flow analysis. *P < 0.05, compared to 30 μM U0126 alone, at respective time points. **E.** Suit-2 and Suit-2^T27N^ cells were treated with 1 μM GEM and cell proliferation was determined after 24, 48, and 72 h. **F.** Cell death in Suit-2 and Suit-2^T27N^ cells treated with 1 μM GEM was assessed with flow analysis. *P < 0.005, compared to Suit-2. Results are presented as mean ± SD. Experiments were repeated thrice, in duplicate.

We next investigated if the release of MVs is critical for the pancreatic cancer cells to resist GEM. Release of MVs is extracellular signal-regulated kinase (ERK) dependent and enabled by ARF6-GTP-dependent recruitment of ERK to the plasma membrane for ERK activation [24]. We inhibited ERK activation using a pharmacological (MEK inhibitor, U0126) inhibitor and a genetic mutation (expression of dominant negative mutant of ARF6, ARF6 T27N) in cell lines that exhibit highest resistance to GEM (Suit-2 and MPanc-96). Cell proliferation and cell death were assessed in these two cell lines after treatment with GEM in the presence or absence of U0126. After 72 h, there was ~ 50% reduction in Suit-2 proliferation upon treatment with U0126 alone, 40% reduction with GEM alone, and 30% reduction with a combination of U0126 and GEM (Figure 4A) with statistical significance (P < 0.05). A similar trend was observed in MPanc-96 cells (Figure 4B). Death assessment at 72 h in Suit-2 cells showed an 11-fold increase in cell death upon combinatorial treatment with U0126 and GEM, compared to control, while the cell death was only 2-fold and 6-fold when treated with GEM alone or U0126 alone, respectively (Figure 4C). Though not to the same degree, combinatorial treatment with U0126 and GEM increased the cell death in MPanc-96 cells compared to control and single treatments (Figure 4D).

To inhibit ERK activation via a genetic mutation, we generated Suit-2^T27N^ cells that stably express the dominant negative mutant of ARF6 to disrupt ERK recruitment to plasma membrane for activation. Inhibition of ERK phosphorylation—and as a consequence MV-release—in Suit-2^T27N^ cells was confirmed (Supplemental Figure S10). Suit-2^T27N^ cells were treated with 1 μM GEM and assessed for cell proliferation and cell death. Cell proliferation was significantly reduced in Suit-2^T27N^ cells compared to parental Suit-2 cells at 48 and 72 h (Figure 4E). At 72 h, compared to untreated cells, parental Suit-2 cells showed a 5-fold reduction and Suit-2^T27N^ cells exhibited a 7-fold reduction in proliferation. When analyzed for cell death, the increase observed in Suit-2^T27N^ cells was significantly higher compared to parental cells at all time points (P < 0.05) (Figure 4F). Thus, the release of MVs is crucial for pancreatic cancer cells to resist drugs and therefore inhibiting their release sensitizes drug-resistant pancreatic cancer cells to GEM *in vitro*.

### Inhibition of MV-release sensitizes GEM-resistant pancreatic cells to GEM *in vivo*

**Figure 5:**
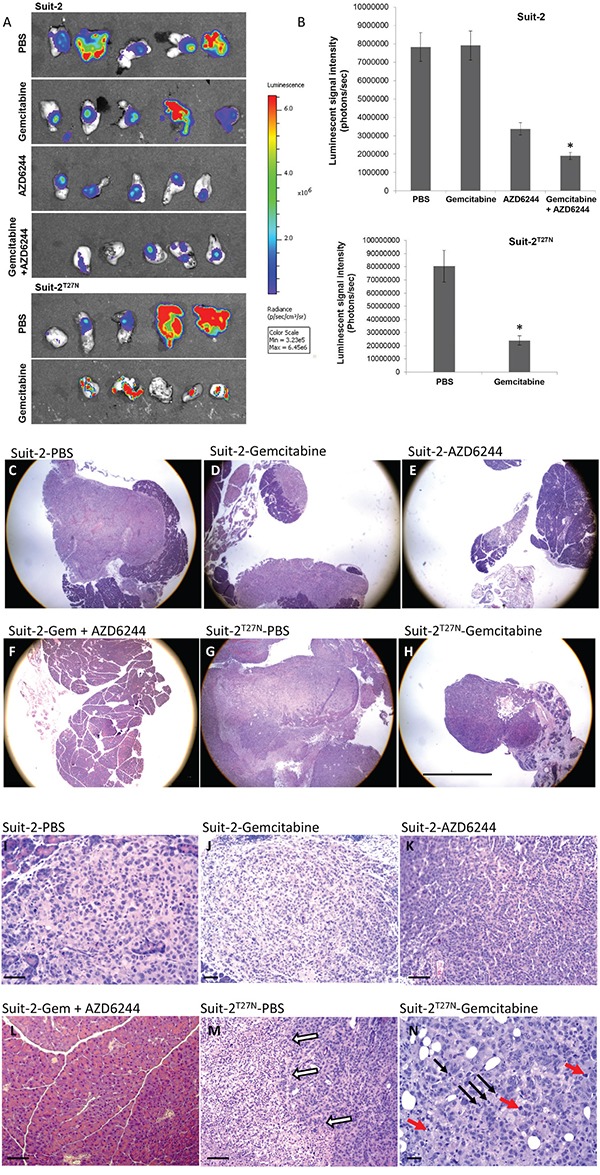
Inhibition of MV-release sensitizes orthotopically implanted GEM-resistant pancreatic tumors to GEM **A.** Bioluminescence imaging of tumors from each group. **B.** Quantification graphs. *P < 0.005 compared to AZD6244 (Suit-2 tumor, top panel) or PBS-treated group (Suit-2^T27N^ tumor, bottom panel). Results are presented as mean ± SE. Representative **C-H.** low magnification and **I-N.** high magnification histopathology images from each group. C, E, G and H show larger tumor masses with extensive central necrosis. D shows a small tumor mass at the edge of the pancreas and a smaller tumor in the same organ. Most of the transplanted tumors are made up of small epithelial cells that grow in solid masses as seen in I and J and sometimes a slight glandular pattern as shown in K. Sections in F and L show an essentially normal pancreas. White arrows in M indicate the border between viable tumor on the right and necrosis on the left. In N the red arrows point to apoptosis and the black arrows to mitoses. The scale bar represents 20 mm. The scale bars for I, K, L, and M are 3 times larger and in N is 4 times larger.

To confirm if inhibiting MV-release also can sensitize pancreatic tumor growth *in vivo*, tumors developed from orthotopically implanted Suit-2-luc or Suit-2^T27N^-luc cells in mice were treated with GEM in the presence and absence of a MEK inhibitor, AZD6244. At the end of treatment, the difference in tumor size based on viable cells present in the tumor was compared using the luminescence. There was a 4-fold decrease in the luminescence of Suit-2 tumors treated with both GEM and AZD6244 compared to the PBS-treated mice (Figure 5A, 5B), and Suit-2 tumors treated with AZD6244 alone had only ~ 2-fold decrease compared to the PBS-treated group. The Suit-2^T27N^ tumors treated with GEM alone showed a 4-fold decrease in luminescence compared to PBS-treated group (Figure 5B).

Reduction of phospho-ERK levels in Suit-2 tumors from mice treated with AZD6244 and Suit-2^T27N^ tumors was confirmed by western analysis (data not shown). The other half of the pancreas was processed and stained for histological analysis. In general the histologic findings confirmed the gross observations. The transplanted tumor cells form fairly discrete masses of undifferentiated adenocarcinoma cells within the pancreas. Although there is a clear demarcation between tumor and uninvolved pancreas in most lesions, occasionally there were strands of tumor cells invading the pancreas for a short distance in the large tumors. Tumor size varied greatly among treatment groups as measured with a stage micrometer (Supplemental Table S1 and Figure 5C-5H). A portion of the tumor was analyzed using histological stain (Figure 5I-5N). The Suit-2 tumors from the GEM-treatment group appear to have a smaller volume than Suit-2 tumors from the PBS-treated group, even though bioluminescence analysis showed no difference between the tumor sizes within these two treatment groups. However, the group treated with both AZD6244 and GEM showed complete absence of any tumor, such that the pathologist scored them as ‘normal pancreases’ (Figure 5L). The Suit-2^T27N^ tumors from mice treated with GEM were made up of small cells with great nuclear variation from small mononuclear to larger multinucleated cells with large nucleoli, with areas containing many mitoses (black arrows, Figure 5N) and apoptotic cells (red arrows, Figure 5N). Analysis of necrotic regions on the tissue sections followed the trend similar to tumor volume analysis (Supplemental Table S1). Tumors from the combination treatment group showed no necrosis. The larger Suit-2^T27N^ tumors treated with PBS had large areas of necrosis (Figure 5G, 5M). The percentage of necrotic tissues decreased by ~2-fold in GEM-treated-Suit-2^T27N^ tumors compared to the PBS-treated group.

Thus, the release of MVs is important for pancreatic cancer cells to resist drugs, and therefore inhibiting their release sensitizes drug-resistant pancreatic cancer cells to GEM *in vivo*.

### Internalized GEM is packaged into MVs before MV-release from cells

We next determined the source of GEM that is removed by the MVs. Is it the GEM that gained entry into cells that gets trafficked into MVs before their release, or is it the GEM in the microenvironment that enters MVs after MV-release, or both?

**Figure 6:**
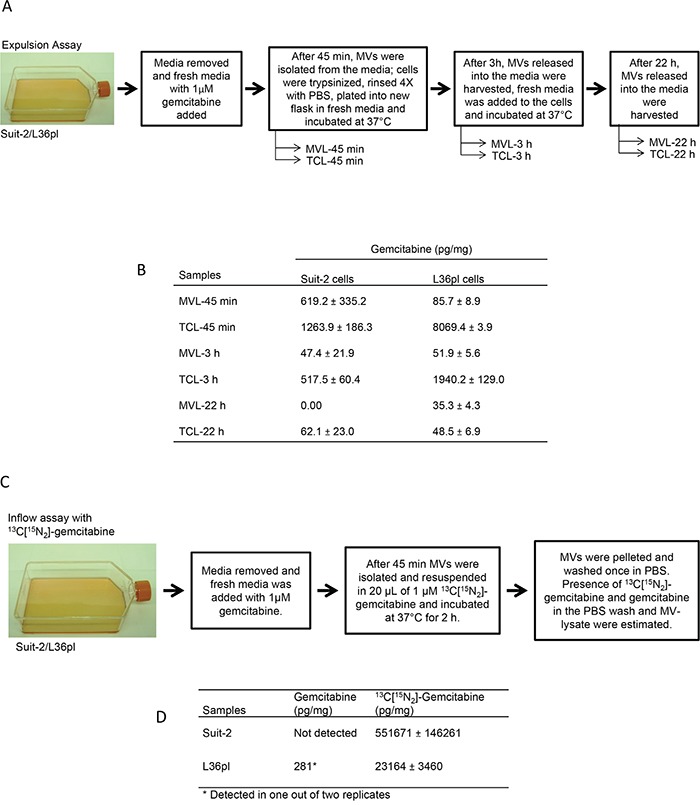
Microvesicles enable removal of drugs from the cells (expulsion assay) and from the microenvironment (inflow assay) **A.** Experimental illustration for expulsion assay. **B.** The amount of GEM (expressed as pg/mg) present in MVL and TCL was measured with LC-MS/MS at the time points indicated. Experiments were repeated thrice. Estimation of drugs in ng/mL is in Supplemental Fig. 11. **C.** Experimental illustration for inflow assay. **D.** The presence of both GEM and ^13^C[^15^N_2_] GEM was analyzed with LC-MS/MS. Experiments were repeated twice, in duplicate and results are presented as mean ± SD. Estimation of drugs in ng/mL is in Supplemental Fig. S12A. TCL = total cell lysates; MVL = MV-lysates.

We did an expulsion assay to determine if GEM that gains entry into the cells gets packaged into MVs (Figure 6A). Both MVs and total cell lysates of Suit-2 and L36pl contained GEM after 45 min (Figure 6B & Supplemental Figure S11). In L36pl cells there was a continued presence of GEM in the MVs released after 45 min, 3 h, and 22 h, indicating that the GEM that gained entry into cells continued to be removed via the released MVs. In contrast, in Suit-2 cells, although GEM was detected in the cell lysates after 45 min, 3 h, and 22 h, GEM was found in MVs only at 45 min and 3 h, but not at 4, 6, or 22 h (4 and 6 h data not shown). The presence of GEM in MVs released by cells (exposed to GEM) in a GEM-free microenvironment indicates that GEM that gained entry into cells was removed via MVs.

### GEM in the microenvironment is packaged into MVs after MV-release from cells

We did an inflow assay to determine if GEM present in the microenvironment can also gain entry into MVs (Figure 6C). MVs released by both Suit-2 cells and L36pl contained ^13^C[^15^N_2_]-GEM, confirming that drug from the microenvironment can enter released MVs (Figure 6D & Supplemental Figure S12A). Analytical details for ^13^C[^15^N_2_]-GEM are in Supplemental Figure S12B. However, there was no GEM detected in MVs released by Suit-2 cells, and GEM was detected in MVs released by L36pl cells in only one of two trials. Thus, GEM in the microenvironment can gain entry into MVs after MV-release.

### GEM treatment specifically increases the amount of influx and efflux protein on MVs

**Figure 7:**
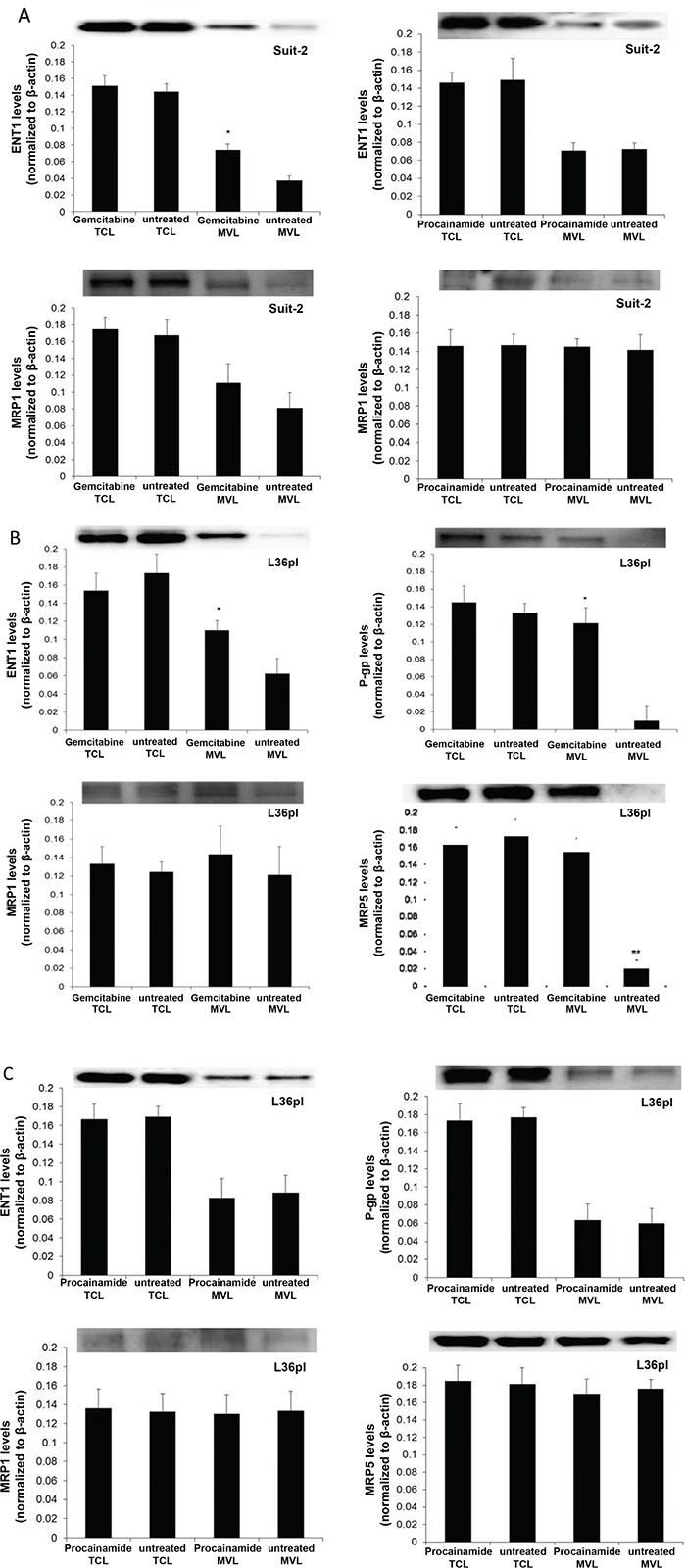
GEM treatment increases expression of efflux and influx proteins on MVs **A.** MVs were isolated from Suit-2 cells treated with GEM or procainamide. Equal amounts of lysates from MVs and cells were tested for ENT1 and MRP1. MVs were isolated from L36pl cells treated with GEM **B.** or procainamide **C.** Equal amounts of lysates from MVs and cells were tested for ENT1, MRP1, P-gp, and MRP5. All the blots were re-probed for β-actin for normalization and graphed. Experiments were repeated thrice. The mean value is plotted as ± SD. *P<0.05, **P<0.005 compared to untreated. MVL= microvesicle lysates; TCL= total cell lysates. The blots showing the relevant bands have been cropped to minimize the blot size.

GEM is a drug that requires transporter proteins to cross cell membrane [25]. Since GEM in the microenvironment also gained entry into the released MVs, we hypothesized that MVs contain transporter proteins. To test this, MVs were isolated from Suit-2 and L36pl cells treated with either GEM or procainamide. The presence of transporter proteins relevant to GEM, namely, influx protein ENT1 and efflux proteins MRP1, MRP5, and P-gp, was analyzed in the lysates. Upon GEM exposure, Suit-2 cells did not exhibit any significant differences in the efflux proteins MRP5 or P-gp (not detected, data not shown) and MRP1 (Figure 7A). While there was no change in ENT1 levels in the cell lysates, levels increased by 2-fold in the MVs released by cells treated with GEM compared to MVs released by untreated cells in both Suit-2 (Figure 7A) and L36pl (Figure 7B). In L36pl cells, GEM exposure did not change MRP1 levels. However, there was a 7-fold increase in the P-gp levels and an 8-fold increase in the MRP5 levels in the MVs released by GEM-treated cells compared to MVs released by untreated cells; there was no significant change in their expression in total cell lysates (Figure 7B). Procainamide treatment did not affect the expression of transporter proteins in Suit-2 cells (Figure 7A), L36pl cells (Figure 7C), or MVs. Thus, collectively GEM exposure increases the levels of ENT1 (influx protein) on the MVs released by both Suit-2 and L36pl cells and increases the levels of P-gp and MRP-5 (efflux proteins) only on the MVs released by L36pl (GEM-sensitive cell line) and not Suit-2 (GEM-resistant cell line).

### GEM is exported by MVs released by L36pl cells but not by MVs released by Suit-2 cells

**Figure 8:**
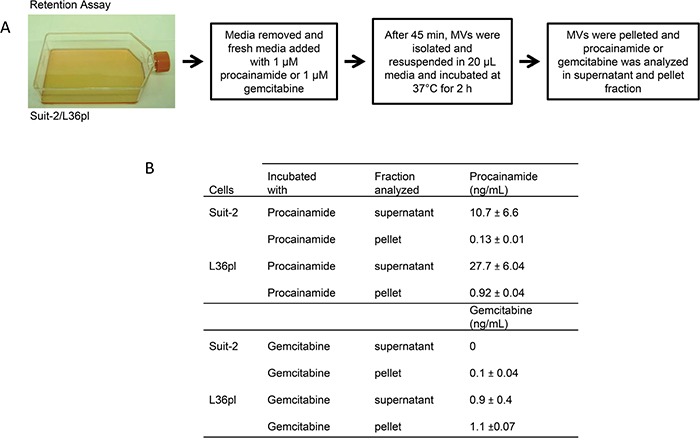
GEM in Suit-2-MVs remains trapped, and GEM in L36pl-MVs flows back to the microenvironment (retention assay) **A.** Suit-2 and L36pl cells were treated as per the illustration. **B.** The amount of GEM or procainamide present in the supernatant or pellet was estimated with LC-MS/MS and expressed in ng/mL. The experiments were repeated thrice, in duplicate. See Supplemental Fig. S13 for the amount of drugs expressed in pg/mg of total protein.

The increase in the efflux proteins on MVs released by L36pl cells upon GEM treatment and not by Suit-2 cells led us to hypothesize that GEM can flow out of the MVs when released by L36pl cells but is trapped in the MVs when released by Suit-2 cells. To test this, we performed a retention assay (Figure 8). While GEM flowed out of the MVs released by L36pl cells into the supernatant, there was no flow of GEM from the MVs released by Suit-2 cells (Figure 8 & Supplemental Figure S13). Procainamide was exported out of the MVs released by both Suit-2 and L36pl cells, indicating the specificity of transporter proteins in the transport of GEM.

## DISCUSSION

Although chemotherapy is a primary strategy to treat cancer in patients, the ability of cancer cells to resist chemotherapy can cause the treatment to fail. It is therefore important to have a comprehensive understanding of mechanisms exploited by cancer cells to resist drug actions in order to develop efficient therapeutic strategies.

Here, we show for the first time that release of MVs by pancreatic cancer cells enables drug resistance by removing anticancer drugs that have entered the cells and that are in the microenvironment. We also elucidated differences between MVs released by drug-resistant and drug-sensitive cells and these are summarized in Table 1.

**Table 1:** Differences between the composition and function of MVs released by drug-resistant Suit-2 and drugsensitive L36pl pancreatic cell lines

	Drug-resistant cell line (Suit-2)	Drug-sensitive cell line (L36pl)
Amount of MVs released in response to GEM	High	Low
GEM clearance in MVs	Cleared by 4 h	Lasts till 22 h
Expression of efflux proteins on MVs	None	Increased
Fate of GEM in MVs	Trapped	Flowed back to microenvironment

In prior growth inhibition studies performed in pancreatic cancer cells, AZD6244 was ineffective *in vitro*, but effective *in vivo* [26–29]. We were therefore limited to using U0126 *in vitro* and using AZD6244 *in vivo*. The combinatorial treatment eradicated Suit-2 tumor cells and restored normal pancreas anatomy. However, it did not completely eradicate Suit-2^T27N^ tumor cells, which could be due to larger initial Suit-2^T27N^ tumor size compared to Suit-2 tumors for the treatment period. There is no known link between ARF6 and trafficking/translocation of transporter proteins for therapeutic drugs. Although stable expression of ARF6-T27N has been shown to affect tumor cell invasion, it does not alter the cell cycle progression because it caused only a small increase in the number of cells in the G2-M phase in melanoma cells [30]. Nonetheless, GEM treatment reduced viable Suit-2^T27N^ tumor cells by 4-fold compared to PBS-treated Suit-2^T27N^ tumors, and a similar fold reduction was seen in Suit-2 tumors treated with AZD6244 + GEM (Figure 4B). A recent study published by Vena et al. [31] also showed that the efficacy of GEM treatment was enhanced in pancreatic cancer models when administered in combination with a MEK1/2 inhibitor, Pimasertib. This study proposed a mechanism independent of MV-release for the increased efficacy. Thus, it cannot be excluded that MEK inhibition can also affect the survival of pancreatic cancer cells independently of MVs [32–34].

The presence of control drugs (ampicillin and procainamide) within MVs released by pancreatic cancer cells suggests that cancer cells may not exert structural or functional specificity in drug-expulsion via MVs. In this context, it is tempting to speculate that normal cells also may employ MVs to buttress the role of P-gp in toxic agent expulsions. The estimation of drugs within MVs is calculated per mg of protein lysates of MVs and not per MV, thus is only semi-quantitative and is a limitation of this study.

In addition to confirming that the GEM that entered cells was removed via MVs, the expulsion assay also revealed differences in GEM clearance in MVs between drug-resistant and drug-sensitive cell lines. While there was a ~10-fold difference in the amount of GEM in MVs released by Suit-2 cells between 45 min and 3 h, there was only a ~2-fold difference in the amount of GEM in the MVs released by L36pl cells (Figure 6A). Because we measured only GEM and not its metabolites, we suspect that the difference in GEM clearance could be due to differences in the levels or activity of GEM-metabolizing enzymes in MVs released by these cells.

The absence of GEM in the MVs released by Suit-2 cells as observed in the inflow assay can be explained by increased GEM clearance, given that the GEM in the MVs released by Suit-2 cells is not detectable even after 4 h (expulsion assay). In the inflow assay, approximately 5 h had elapsed (2 isolations of MVs for ~3 h + 2 h of incubation) by the time the MVs were isolated for analysis. It is, however, intriguing that only trace levels of GEM were detected (281 pg/mg) in one of the two replicates in MVs released by L36pl cells, whereas GEM was detected in MVs from L36pl cells even after 22 h (expulsion assay). This can be explained by the presence of high levels of efflux proteins on L36pl-MVs that enabled displacement of GEM in the MVs by ^13^C[^15^N_2_]-GEM entering from the microenvironment.

Collectively, this study reveals a novel mechanism in which pancreatic cancer cells remove drugs via MVs. In addition, the study also clarifies differences between the MVs released by drug-resistant and drug-sensitive pancreatic cancer cells. The importance of MV-release in the acquisition of drug resistance as revealed here by animal studies has implications for human pancreatic cancer patients and the possibility of overcoming drug resistance.

## MATERIALS AND METHODS

### Materials

Media (DMEM, McCoy5a, RPMI), penicillin, streptomycin, FBS, L-glutamine, G418, along with GEM, doxorubicin, 5-fluorouracil, paclitaxel, procainamide, and ampicillin were purchased from Sigma-Aldrich (St. Louis, MO) and reconstituted as per manufacturer's instructions. Selumetinib (AZD6244) was purchased from ChemieTek (Indianapolis, IN) and reconstituted in 1X PBS at 10 mg/mL concentration, which formed a white, turbid suspension. GEM was dissolved in 1X PBS at 5 mg/mL. U0126 was purchased from Tocris Biosciences (Bristol, UK) and reconstituted in DMSO at 10 mM. ^13^C[^15^N_2_]-GEM (99% purity) was obtained from Moravek Biochemicals (Brea, CA). Methanol, acetonitrile (HPLC grade), formic acid, and acetic acid were purchased from Mallinckrodt Baker (Phillipsburg, NJ). Antibodies against P-gp, ENT1, MRP1, MRP5, β-actin, and secondary antibodies were purchased from Abcam (Cambridge, MA), and antibodies against phospho-ERK and total-ERK were purchased from Cell Signaling (Danvers, MA) and ARF6 from Abgent (San Diego, CA). The antibodies were previously published [35].

### Cell culture

Pancreatic cancer cells MPanc-96, L36pl, Suit-2, BxPc3, and MiaPaCa-2 were obtained from Dr. Thiruvengadam Arumugam (MD Anderson Cancer Center, Houston, TX) and were genotyped with DNA fingerprinting [36]. Breast cancer cells (FMMC 419II) were obtained from MMTV-PyMT mice [23]. MPanc-96 and Suit-2 cells express luciferase gene constitutively. L36pl cells were cultured in RPMI media, Capan-2 cells were cultured in McCoy5a media, and all other cell lines were cultured in DMEM and maintained at 37°C in a 5% CO_2_ humidified incubator. Suit-2^T27N^ cell line that stably expresses a dominant negative mutant of ARF6, ARF6-T27N, was generated by transfection of pCDNA3 HA ARF6 DN T27N plasmid, a gift from Thomas Roberts (Addgene plasmid # 10831) [37], with constant selection in G418 (200 μg/mL).

### MV isolation, quantitation, and size distribution

Equal numbers of cells were plated and media was changed after 24 h with or without drug treatment. MVs were isolated by differential centrifugation [3], first at 150 x *g* for 10 min to remove cells and larger debris, followed by 2,500 x *g* centrifugation for 20 min to pellet larger apoptotic bodies. The supernatant devoid of apoptotic bodies was centrifuged at 12,200 x *g* for 40 min to pellet MVs. Isolated MVs were rinsed once in PBS and resuspended in PBS and quantitated by measuring surface proteins using NanoDrop 2000 (Thermo Scientific, Wilmington, DE). Size distribution of MVs was analyzed at 25°C with dynamic light scattering (DLS) using a Malvern Zetasizer (Malvern Instrumentation Co, Westborough, MA).

### Atomic force microscopy

Isolated MVs from all cell lines were resuspended in PBS. Structural analysis and imaging of MVs were performed using atomic force microscopy (AFM) (MFP 3D, Asylum Research, Santa Barbara, CA), using semi-dry mode of scanning at the Rensselaer Polytechnic Institute Core Facility (Troy, NY). Single crystal high resolution silicon AFM probe from NT-MDT (NSG03) with a rectangle cantilever was used. The AFM probe had a cantilever force constant of around 1.8 N/m, a resonance frequency of 70 kHz, and a tip radius of less than 10 nm. IGOR6 software (WaveMetrics, Portland, OR) was used for image processing.

### MTT proliferation assay

Equal numbers of cells were seeded in 96-well plates and treated with appropriate drugs. After appropriate passage of time, cell proliferation was measured by Vybrant MTT (3(4, 5-dimethylthiazol-2-yl)-2, 5-diphenyltetrazolium bromide) assay as per manufacturer's instructions (Invitrogen, Grand Island, NY). The optical absorbance was read at 540 nm using Synergy 2 plate reader (BioTek, Winooski, VT).

### Immunofluorescence staining

Cultured cells plated on glass coverslips were fixed and processed as described earlier [30]. F-actin distribution was visualized by staining with rhodamine-phalloidin substrate (Thermo Fisher Scientific, Wilmington, DE). Cells were visualized with a Nikon microscope coupled to a Bio-Rad MRC 1024 scanning confocal three-channel system.

### Protein sample preparation and western blotting

Protein lysates from cells were prepared using either CelLytic™ M buffer or RIPA buffer, containing protease inhibitor cocktail and/or phosphatase inhibitor cocktail (Sigma-Aldrich). Equal amounts of proteins were separated by SDS electrophoresis, transferred to PVDF membrane, and probed with appropriate primary and secondary antibody. The signals were developed using SuperSignal West Pico Chemiluminescent Substrate (Thermo Fisher Scientific, Grand Island, NY). Blots were imaged with Chemidoc XRS (Bio-Rad, Hercules, CA) and net band intensity was measured with ImageLab software (Bio-Rad).

### Flow cytometry for apoptosis quantitation

Equal numbers of cells were plated and treated with either 1 or 10 μM GEM. After 24, 48, and 72 h cells were harvested and resuspended in Annexin binding buffer. Apoptotic cells were double stained using an Annexin V-FITC apoptosis detection kit according to the manufacturer's protocol (BD Biosciences, San Jose, CA) [38]. Flow analysis of the stained cells was performed using FACSAria II (BD Biosciences) at the Neural Stem Cell Institute (Rensselaer, NY).

### HPLC-MS/MS analysis of GEM, ^13^C[^15^N_2_]-GEM, 5-fluorouracil, ampicillin, and paclitaxel

Total cells and isolated MVs were lysed in CelLytic™M buffer. A dilute-and-shoot protocol was used for analyzing samples. Sample (12.5 μL) was transferred into a 15-mL polypropylene tube, and 12.5 μL of acetonitrile:Milli-Q water (1:9, % v/v) was added, vortex mixed, and transferred for HPLC–MS/MS analysis. Standard solutions were prepared in acetonitrile:Milli-Q water (1:9, % v/v). Identification and quantification of GEM, ^13^C[^15^N_2_]-GEM, 5-fluorouracil, ampicillin, and paclitaxel in lysates, PBS wash, media, and control samples were performed with an Applied Biosystems API 2000 electrospray triple quadrupole mass spectrometer (Applied Biosystems, Foster City, CA) at a resolving power of 0.70 FWHM. Individual parameters to measure GEM, ^13^C[^15^N_2_]-GEM, 5-fluorouracil, ampicillin, and paclitaxel are provided in detail in Supplemental Information.

### Animal studies

NCr male nude mice aged 5–6 weeks were purchased from Harlan Laboratories (Indianapolis, IN) and maintained under recommended, controlled conditions with *ad libitum* access to water and food. All animal studies were conducted at the animal facility of the Veteran Affairs Medical Center, Albany, NY, in accordance with the institutional guidelines for humane animal treatment and according to the current NIH guidelines. Suit-2-luc cells and Suit-2^T27N^-luc cells were orthotopically implanted (2 × 10^5^ cells in 50 μL PBS per mouse) in the pancreas of anesthetized mice. Prior to initiating treatments, animals (n = 5 or 6 per group) were randomized according to the signal intensity of tumors imaged with an *in vivo* imaging system (IVIS, described in Supplemental Information). GEM at 25 mg/kg was injected intraperitoneally twice a week, and AZD6244 [39] was administered orally, daily at 50 mg/kg. There were 4 treatment groups for Suit-2 tumors (PBS, AZD6244, GEM, and GEM + AZD6244) and 2 treatment groups for Suit-2^T27N^ tumors (PBS and GEM). After 28 days of treatment, pancreases along with tumors were collected and imaged for bioluminescent signal intensity and weighed. Samples were frozen for molecular analysis, and a portion was fixed for histopathological studies.

### Histopathology

Sections from formalin-fixed specimens were processed and stained routinely for analysis. Individual sections (2 per animal) were blinded for identity and presence of tumor, and necrosis was scored by a board certified pathologist (S. S.). The tumor size was obtained by measuring the cross diameters using a stage micrometer. Percentage of necrosis in tumor mass and number of cells undergoing mitoses and apoptosis were estimated per high powered field. Sections were imaged using an Olympus BX51 light microscope with a model U-LH100HG digital camera.

### Statistical analysis

Analysis of the *in vivo* study results was done with one-way ANOVA using StatView software (Adept Scientific, Acton, MA). For the *in vitro* studies, the unpaired t-test was used for analysis.

## SUPPLEMENTARY MATERIALS FIGURES


