# Supplementary material for: Microvesicle removal of anticancer drugs contributes to drug resistance in human pancreatic cancer cells
Source: Oncotarget. 2016 Jul 4;7(31):50365–79. doi: 10.18632/oncotarget.10395 (PMC5226588; doi:10.18632/oncotarget.10395)
Supplement: Supplementary file 1 [file oncotarget-07-50365-s001.pdf]

Microvesicle removal of anticancer drugs contributes to drug resistance in human pancreatic cancer cells

SUPPLEMENTARY DATA

METHODS

Flow cytometry for doxorubicin detection and quantitation

FMMC breast cancer cells were treated for 3 h with doxorubicin (1 µg/mL) and MVs were released from both treated and untreated cells. Analysis of the accumulated doxorubicin within the cells and isolated MVs was carried out in FL2-H channel. Flow analysis of the stained cells was performed using FACSAria II (BD Biosciences) at the Neural Stem Cell Institute (Rensselaer, NY).

In vivo imaging system (IVIS)

Imaging was performed once per week to monitor tumor growth. Mice bearing Suit2-luc cells and Suit-2^{T27N}-luc cells were anaesthetized using isoflurane and injected subcutaneously with 50 µL of D-luciferin (30 mg/mL) prior to imaging. Photographic and luminescence images were taken at constant exposure time using a Xenogen IVIS imager (PerkinElmer, Waltham, MA). Living Image software version 3.2 was used to quantify non-saturated bioluminescence in regions of interest. Bioluminescence was quantified as photons/s and light emission between 5.5 x 10^6 and 7 x 10^{10} is indicative of viable luciferase-labeled tumor cells, while emissions below this range were considered as background.

UPLC-MS/MS analysis and drug uptake determination of gemcitabine and procainamide

Chromatography was performed with a Waters ACQUITY UPLC system (Waters, Milford, MA). The column was an ACQUITY UPLC BEH C18 (130Å, 1.7 µm, 2.1 mm X 50 mm; Waters) serially connected to a VanGuard™ Pre-Column (BEH C18, Waters). The mobile phase was 0.1% v/v formic acid in water (A) and acetonitrile (B); this mobile phase gave optimal peak shape and instrumental response. A gradient elution was used at a flow rate of 100 µL/min starting at 90% (v/v) A and 10% B, held for 1.5 min, followed by a linear gradient to 90% B and 10 % A over 1.5 min, after which the system was reverted to the initial condition in 30 seconds. The standard solutions in CelLytic™ M (Sigma-Aldrich) were prepared in the range 0.25-20 ng/mL (r^2 > 0.99) concentration. For the uptake study, 10 µL of samples (the culture media or lysates from total cells or microvesicles lysed in CelLytic™ M buffer) were injected directly into the UPLC. Gemcitabine (gem) or procainamide concentrations were determined by calculating the area under the eluted peak and comparing to a standard curve. The amount of gem or procainamide uptake in picogram per mg protein was reported.

Identification and quantification of target analytes was performed with an ACQUITY® TQD tandem quadrupole MS under positive ionization mode. The cone voltage was set at 30 kV, curtain and collision gas (nitrogen) flow rates were 10 and 2 psi, respectively, and the source heater was set at 150°C. The nebulizer gas (ion source gas 1) was 50 psi, and the heater gas (ion source gas 2) was 55 psi. Data acquisition scan speed was 195 ms with a resolving power of 0.70 FWHM. Injection volume was 10 µL. The instrumental limits of detection (LODs) and limits of quantification (LOQs) were the same for both gem and procainamide. The instrumental LOD was obtained as 3.3 times the standard deviation of the calibration curve divided by the slope of the curve. The LOQ was set as 3 times higher than the LOD. Thus, the instrumental LOD and LOQ were 0.125 and 0.375 ng/mL, respectively. Data were acquired with Empower software (Waters). Multiple reaction monitoring (MRM) settings for gem and procainamide were 264.04 → 112.0 and 236.11→163.01, respectively.

HPLC-MS/MS analysis of gemcitabine, $^{13}C[^{15}N_2]$-gemcitabine, 5-fluorouracil, ampicillin, and paclitaxel

Chromatography was performed with an Agilent (Santa Clara, CA) 1100 Series HPLC system. The column was a Zorbax SB-Aq (150 mm X 2.1 mm, 3.5 µm) serially connected to a Javelin guard column (Betasil C18, 2.1 mm X 20 mm, 5 µm, Thermo Electron Corp., Waltham MA). For the measurement of gemcitabine and $^{13}C[^{15}N_2]$-gemcitabine, the mobile phase was 0.1% v/v acetic acid in acetonitrile (A) and 0.1% v/v acetic acid in Milli-Q water (B). A gradient elution was used at a flow rate of 200 µL/min starting at 95% B, held for 1 min (1st min), decreased to 2% B within 17 min (18th min), held for 2 min (20th min), and reverted to 95% B at the 21st min that was then held for 4 min. The mass spectrometry system was an Applied Biosystems (Foster City, CA) API 2000 electrospray triple quadrupole instrument operated in

positive ionization mode. The electrospray ionization voltage was set at +4.7 kV, curtain and collision gas (nitrogen) flow rates were 10 and 2 psi, respectively, and the source heater was set at 550°C. The nebulizer gas (ion source gas 1) was 69 psi, and the heater gas (ion source gas 2) was 68 psi. Multiple reaction monitoring (MRM) settings for gem and $^{13}C[^{15}N_2]$-gem were 264.1 → 111.9 and 267.1 → 114.9 m/z, respectively. The LODs and LOQs for both gem and $^{13}C[^{15}N_2]$-gem were 0.03 and 0.1 ng/mL, respectively.

For the measurement of 5-fluorouracil, the HPLC mobile phase was the same as stated above for gem. A gradient elution was used at a flow rate of 200 µL/min starting at 70% B, held for 3 min (3rd min), decreased to 5% B within 7 min (10th min), held for 0.5 min (10.5th min), and reverted to 70% B at the 15th min that was then held for 3 min. The electrospray ionization voltage was set at -3.0 kV. The curtain and collision gas (nitrogen) flow rates were set at 10 and 4 psi, respectively, and the source heater was set at 550°C. The nebulizer gas (ion source gas 1) was set at 60 psi, and the heater gas (ion source gas 2) was set at 60 psi. MRM settings for 5-fluorouracil were 128.9 → 42.1 m/z (quantification ion) and 128.9 → 59.0 m/z (confirmation ion). The LOD and LOQ were 0.3 and 1 ng/mL, respectively.

For the measurement of ampicillin and paclitaxel, the HPLC gradient was the same as stated above for gem. However, for the measurement of ampicillin, the mobile phase was 0.1% v/v formic acid in methanol (A) and 0.1% v/v formic acid in Milli-Q water (B), and for the measurement of paclitaxel the mobile phase was 0.01% v/v acetic acid in methanol (A) and 0.01% v/v acetic acid in Milli-Q water (B). The tandem MS parameters for ampicillin were the same as stated for gem. The tandem MS parameters for ampicillin differed from that of gem in the collision gas flow rate, which was set at 5 psi, the nebulizer gas (ion source gas 1) at 15 psi, and the heater gas (ion source gas 2) at 64 psi. MRM settings for ampicillin were 350.1 → 106.3 (quantification ion) and 350.1 → 160.0 (confirmation ion) m/z. The LOD and LOQ for ampicillin were 3.6 and 10.8 ng/mL, respectively. MRM settings for paclitaxel were 876.3 → 308.4 ($[M+Na]^+$, quantification ion), 854.1 → 296.1 ($[M]^+$, confirmation ion) and 892.1 → 324.0 ($[M+K]^+$, confirmation ion) m/z. The LOD and LOQ for paclitaxel were 1.7 and 5.1 ng/mL, respectively.

Second confirmation method for gemcitabine and $^{13}C[^{15}N_2]$-gemcitabine

Sample (12.5 or 25 µL) was transferred into a 15-mL polypropylene tube, and 1 mL of acetonitrile was added. The tube was left in a -20°C freezer overnight for freeze precipitation. Thereafter, the supernatant (if the precipitate was obtained) or the whole solution (if the precipitate was not obtained) was collected and filtered through a 15 mm Syringe Filter 0.2 µm (Phenomenex Inc., Torrance, CA). Samples with a formed precipitate were immediately separated from the supernatant after collection from the freezer. The eluent obtained after filtration was transferred into a 15-mL polypropylene tube for concentration to dryness under a gentle nitrogen stream. Finally, 25 µL of acetonitrile:Milli-Q water (1:9, %v/v) was added, vortex mixed, and transferred for HPLC-MS/MS analysis. Throughout the analysis of actual samples, 6 pre-extraction matrix (PBS) spikes were prepared for the analysis by spiking known concentrations of target analytes and passing them through the entire analytical procedure. Recoveries (N=6; ± standard deviation) were 48.3 ± 5.1 %.

A calibration check standard and methanol standard were injected as a check for drift in instrumental sensitivity and carry-over between samples, respectively. The quantification was accomplished with a matrix-matched calibration standard prepared by spiking target analytes into a matrix (PBS-solution) prior to extraction. Samples that presented higher peak area than that of the highest point of the calibration curve were diluted accordingly prior to analysis.

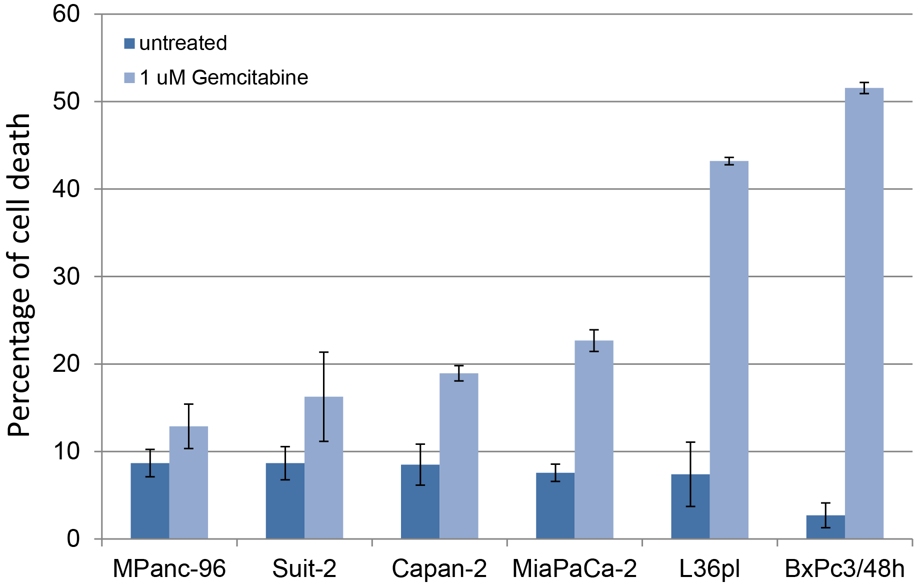

Supplementary Figure S1: Pancreatic cancer cells exhibit varying degrees of resistance to gemcitabine. Cells from each cell line (MPanc-96, Suit-2, Capan 2, MiaPaca-2, L36pl, and BxPc3) were plated and treated with 1 µM gemcitabine. After 72 h, (except BxPc3, after 48 h), cells were double stained with Annexin V-FITC and propidium iodide, according to the manufacturer's protocol (BD Biosciences, San Jose, CA). The apoptotic profile was acquired and analyzed using BD FACS Diva. Data were obtained from 3 independent experiments and presented as percentage mean ± SD.

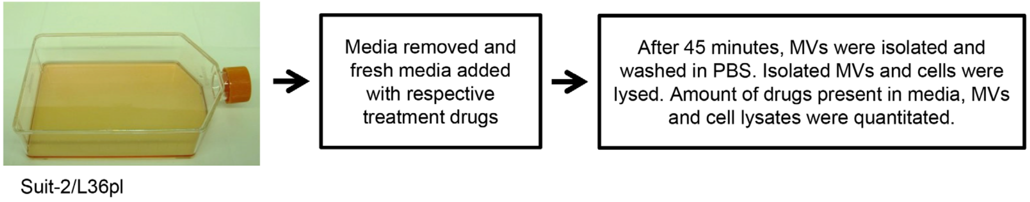

	Suit-2 cells			L36pl cells		
Drug	TCL-45‘ (ng/mL)	MVL-45‘ (ng/mL)	Media-45‘ (ng/mL)	TCL-45‘ (ng/mL)	MVL-45‘ (ng/mL)	Media-45‘ (ng/mL)
Gemcitabine	24.2 ± 1	0.6 ± 0.05	109.9 ± 1.8	16.9 ± 1.6	0.12 ± 0.07	33.0 ± 0.6
5-Fluorouracil	130 ± 21.3	61.5 ± 17.2	20272 ± 3174	123 ± 5.5	1382 ± 159	24480 ± 2678
Paclitaxel	23.5 ± 0.2	90.8 ± 3.5	10 ± 5.8	14.5 ± 4.9	485 ± 357	4 ± 4
Procainamide	0.31 ± 0.2	3.8 ± 0.1	162.2 ± 2.6	0.16 ± 0.01	0.93 ± 0.1	85.8 ± 30
Ampicillin	18.4 ± 10.9	213 ± 40.9	17593 ± 11848	26.4 ± 5.5	423 ± 190	177838 ± 43622

Supplementary Figure S2: Microvesicles contain therapeutic drugs. MVs released by Suit-2 and L36pl cells were isolated as outlined in the illustration after treatment with various drugs. The amount of drugs present in the media, in total cells (TCL), and in released MVs (MVL) was estimated with HPLC-MS/MS and is expressed as ng/mL. The experiments were repeated twice, in duplicate.

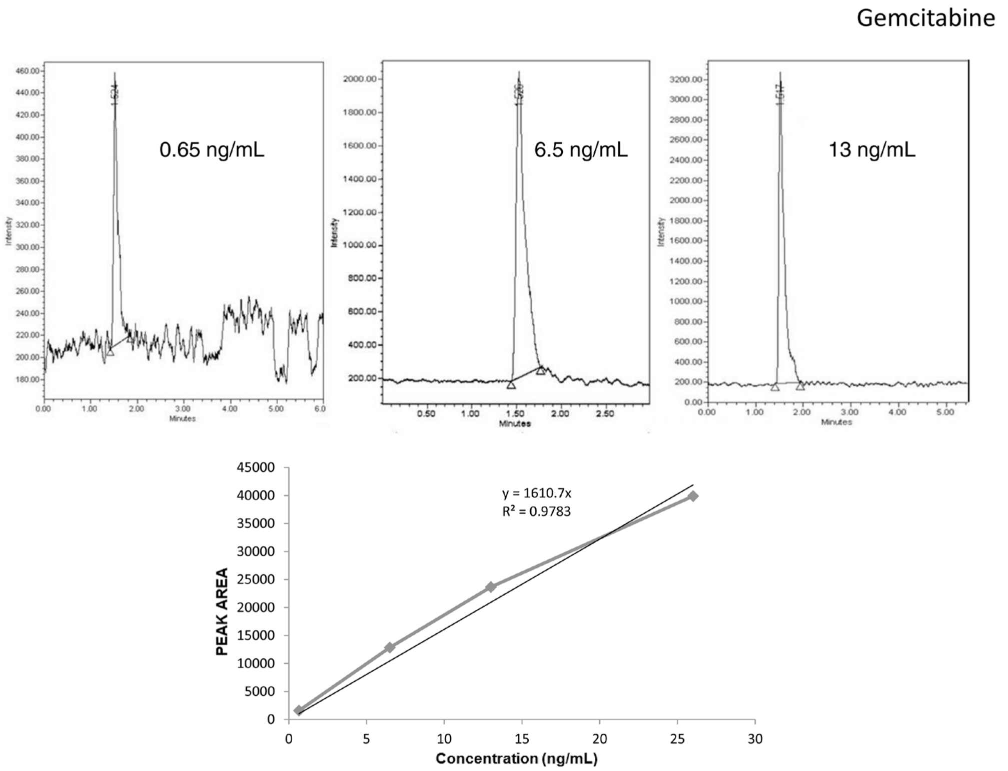

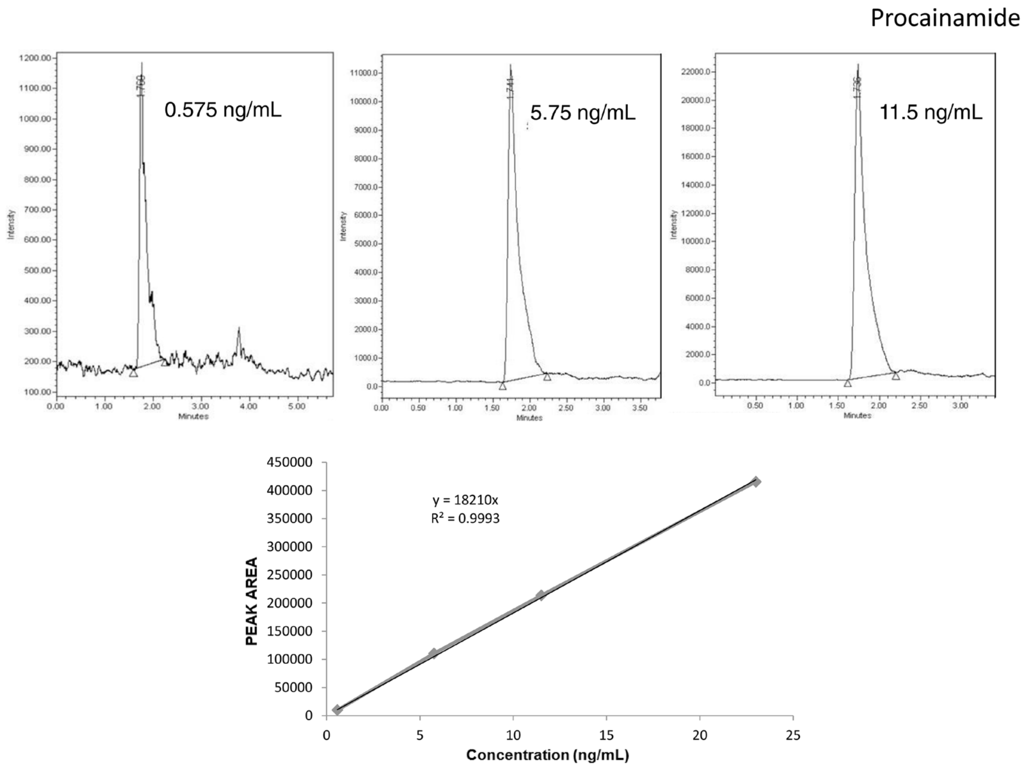

Supplementary Figures S3-S4: Detection and quantitation of gemcitabine (S3) and procainamide (S4) with UPLC. Top Panels:UPLC analysis of gemcitabine and procainamide standards in increasing concentrations (left to right). Bottom Panels:Calibration curves for gemcitabine and procainamide. The graphs shows good linearity indicating the power of gemcitabine and procainamide detection and quantitation with UPLC analysis.

5-Fluorouracil

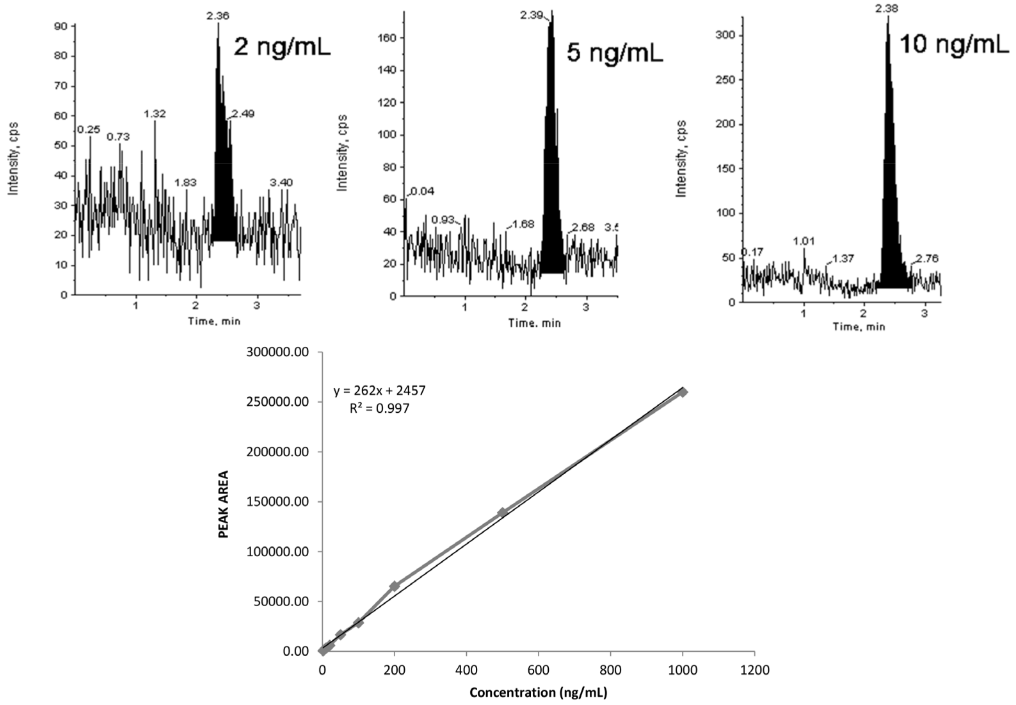

Paclitaxel

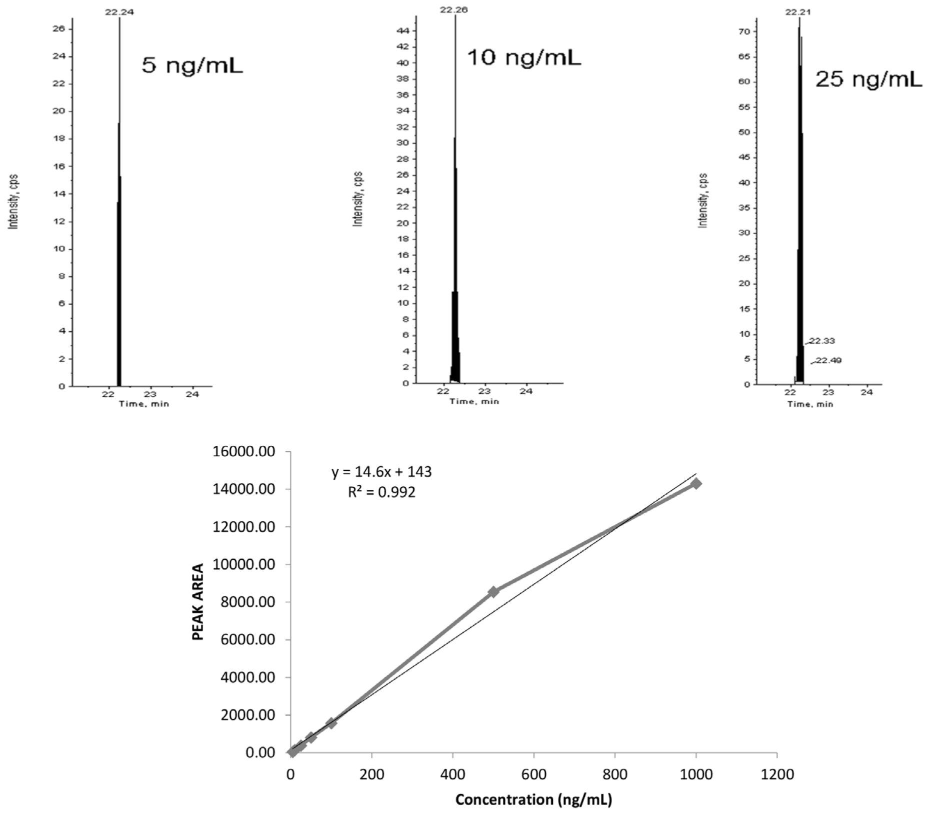

(*Continued*)

Ampicillin

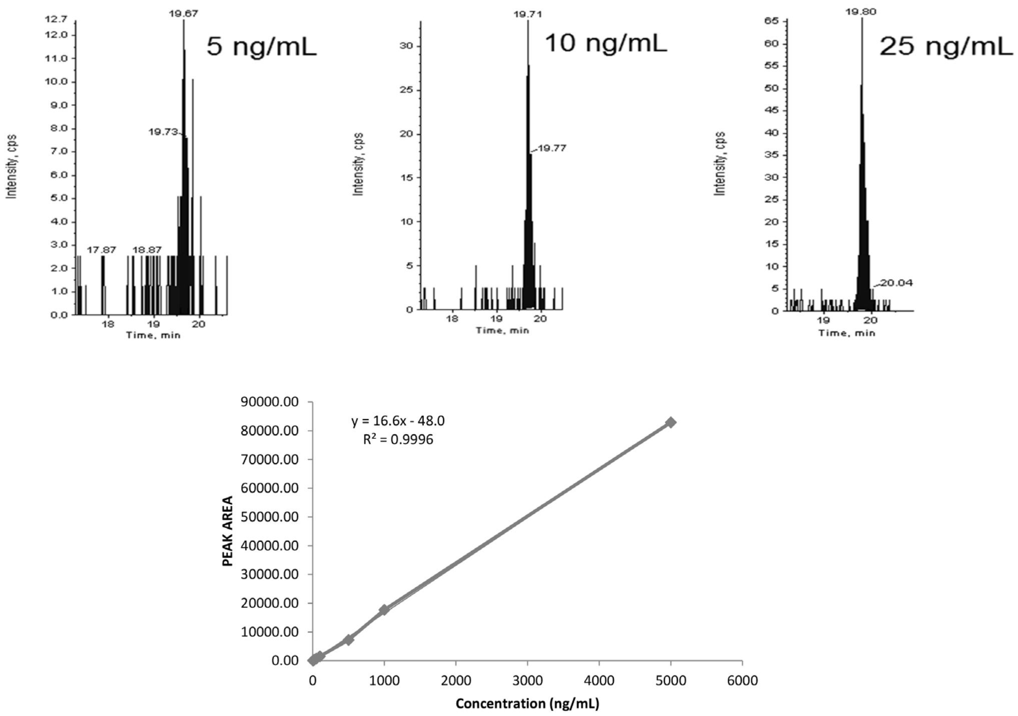

Gemcitabine

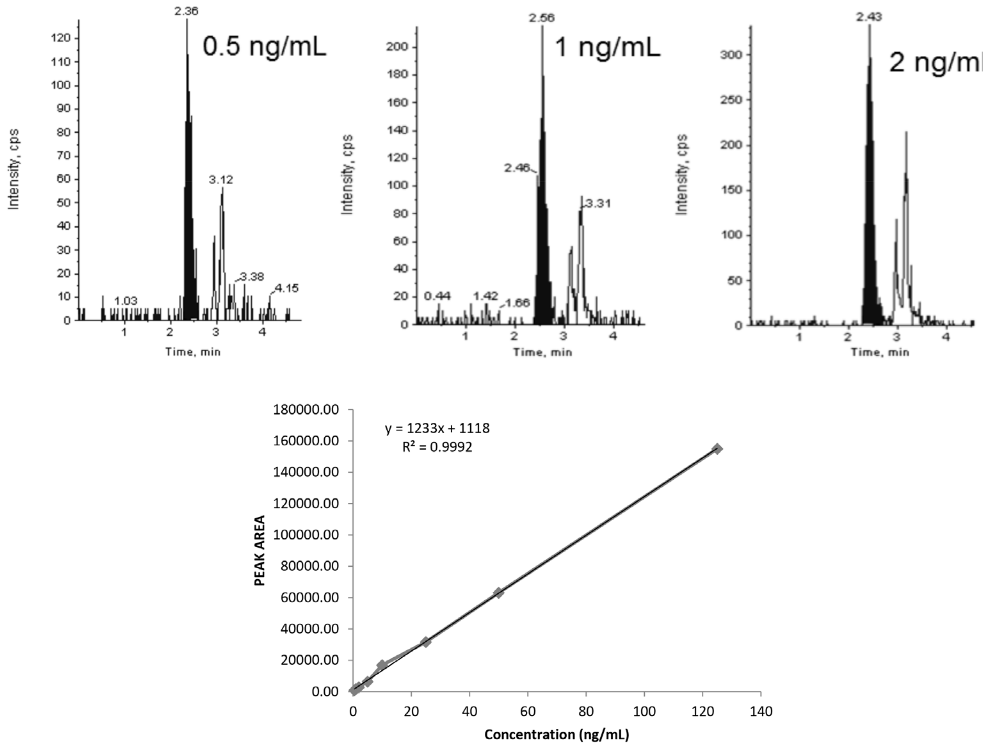

Supplementary Figures S5-S8: Detection and quantitation with HPLC-MS/MS of 5-Fluorouracil (S5), Paclitaxel (S6), Ampicillin (S7), and Gemcitabine (S8). Top Panels:HPLC analysis of respective compound standards in increasing concentrations (left to right). Bottom Panels:Calibration curve generated from respective standards. The graphs shows good linearity indicating the power of detection and quantitation of 5-Fluorouracil, Paclitaxel, Ampicillin, and Gemcitabine with HPLC-MS/MS analysis.

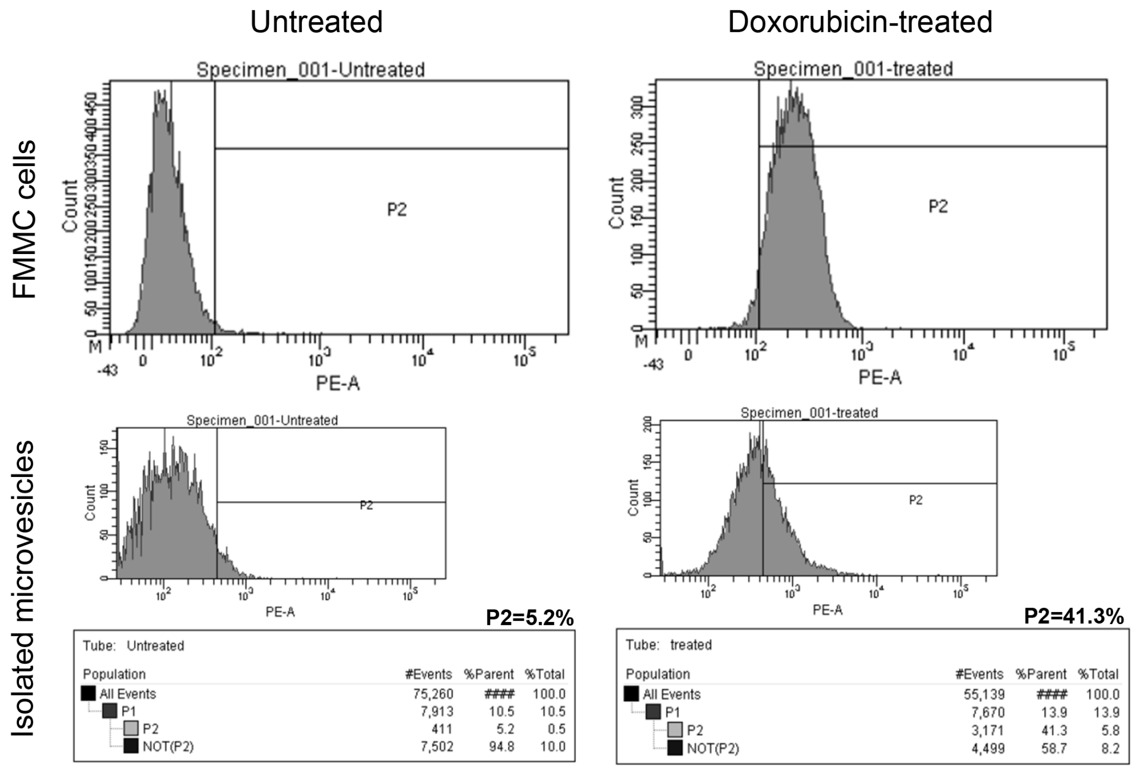

Supplementary Figure S9: Microvesicles released by FMMC breast cancer cells enable expulsion of doxorubicin from cells. FMMC breast cancer cells were treated with 1 µg/mL of doxorubicin for 3 h. Microvesicles were isolated from both untreated and treated cells. Detection of the presence of doxorubicin within the treated and untreated cells (top panel) and microvesicles (bottom panel) was carried out in FL2-H channel. Accumulation of doxorubicin was 8-fold more in the microvesicles isolated from treated cells compared to microvesicles isolated from untreated cells.

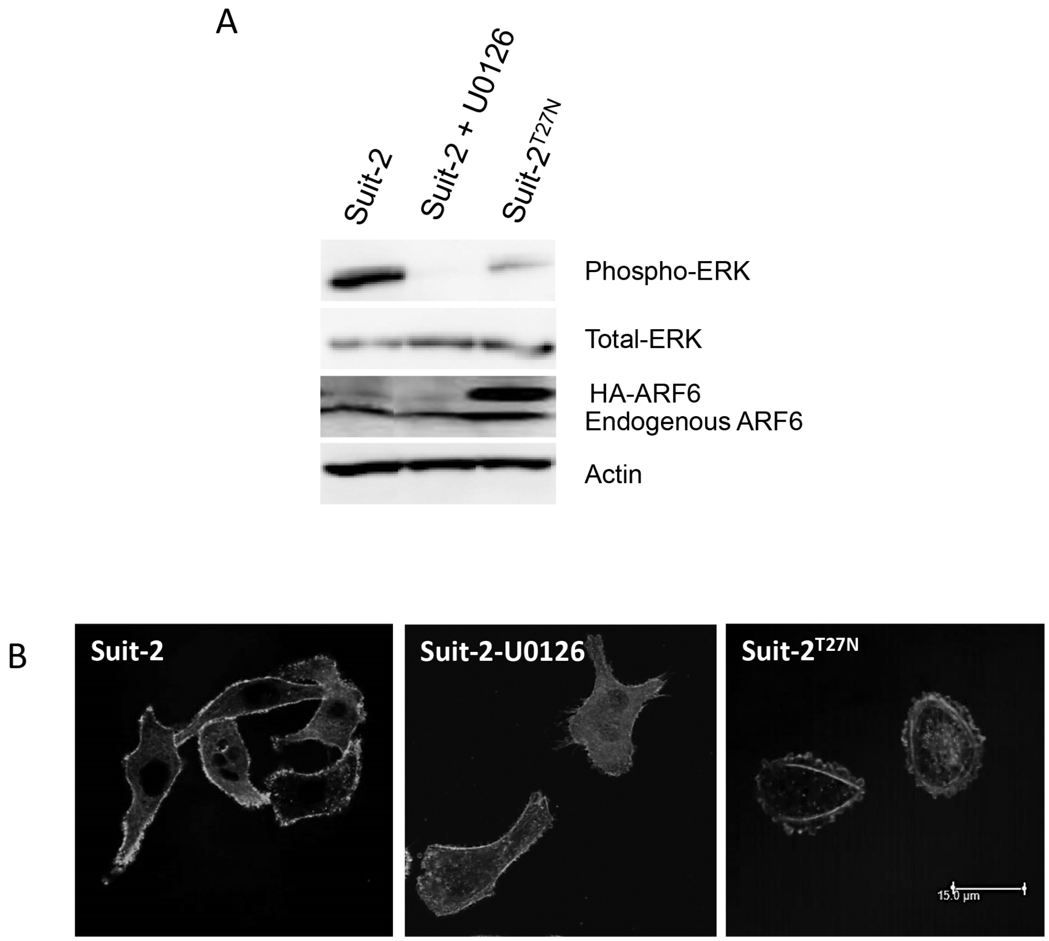

Supplementary Figure S10: Characterization of Suit-2T27N cells. A. Treatment with U0126 and/or ARF6 T27N expression inhibits ERK phosphorylation. Suit-2^{T27N} cell line was generated by stable expression of a dominant negative mutant of ARF6, ARF6-T27N tagged with HA. Suit-2 cells were treated with 30 μM U0126 for 17 h. Total cell lysates from untreated and treated Suit-2 cells and Suit-2^{T27N} cells were probed for the presence of phospho-ERK, Total ERK, ARF6 and Actin. The blots showing the relevant bands have been cropped to minimize the blot size. **B.** Inhibition of ERK phosphorylation results in the inhibition of the release of microvesicles. Suit-2^{T27N} cells and Suit-2 cells were plated on glass coverslips. Suit-2 cells were treated with U0126. Suit-2^{T27N} cells, and treated and untreated Suit-2 cells were fixed, stained with rhodamine-phalloidin, and imaged.

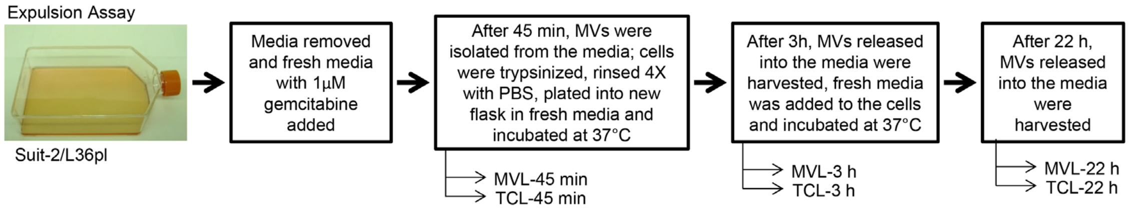

Samples analyzed for gemcitabine	Suit-2 cells (ng/mL)	L36pl cells (ng/mL)
MVL-45 min	0.8 ± 0.5	0.22 ± 0.1
TCL-45 min	1.7 ± 0.2	30.7 ± 0.01
MVL-3 h	0.07 ±0.03	0.1 ± 0.01
TCL-3 h	1.3 ± 0.29	15.3 ± 0.7
MVL-22 h	0.00	0.09 ± 0.01
TCL-22 h	0.4 ± 0.17	0.29 ± 0.04

Supplementary Figure S11: Microvesicles enable expulsion of drugs that entered the cells and removal of drugs from the microenvironment. Gem internalized by cells is expelled via MVs (expulsion assay). Suit-2 and L36pl cells were treated as shown in the illustration and released MVs were isolated. The amount of gem present in MV-lysates (MVL) and total cell lysates (TCL) was measured with HPLC-MS/MS at the time points indicated. The amount of gem detected is expressed as ng/mL in lysates. Experiments were repeated thrice.

A

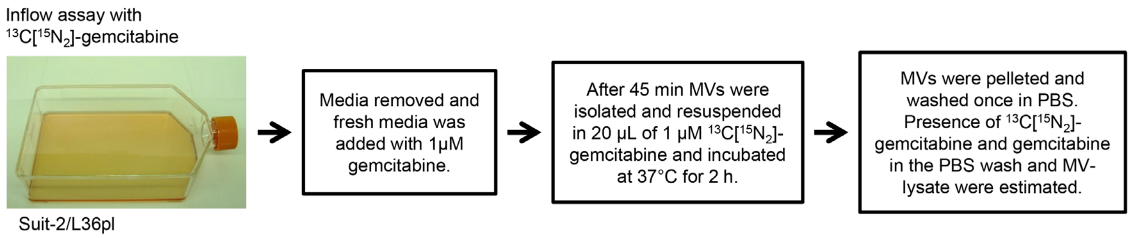

Samples from	Gemcitabine (ng/mL)	$^{13}C[^{15}N_2]$-Gemcitabine (ng/mL)
Suit-2	Not detected	30.9 ± 11..9
L36pl	0.27*	21.4 ± 4.5

* Detected in one out of two replicates

B

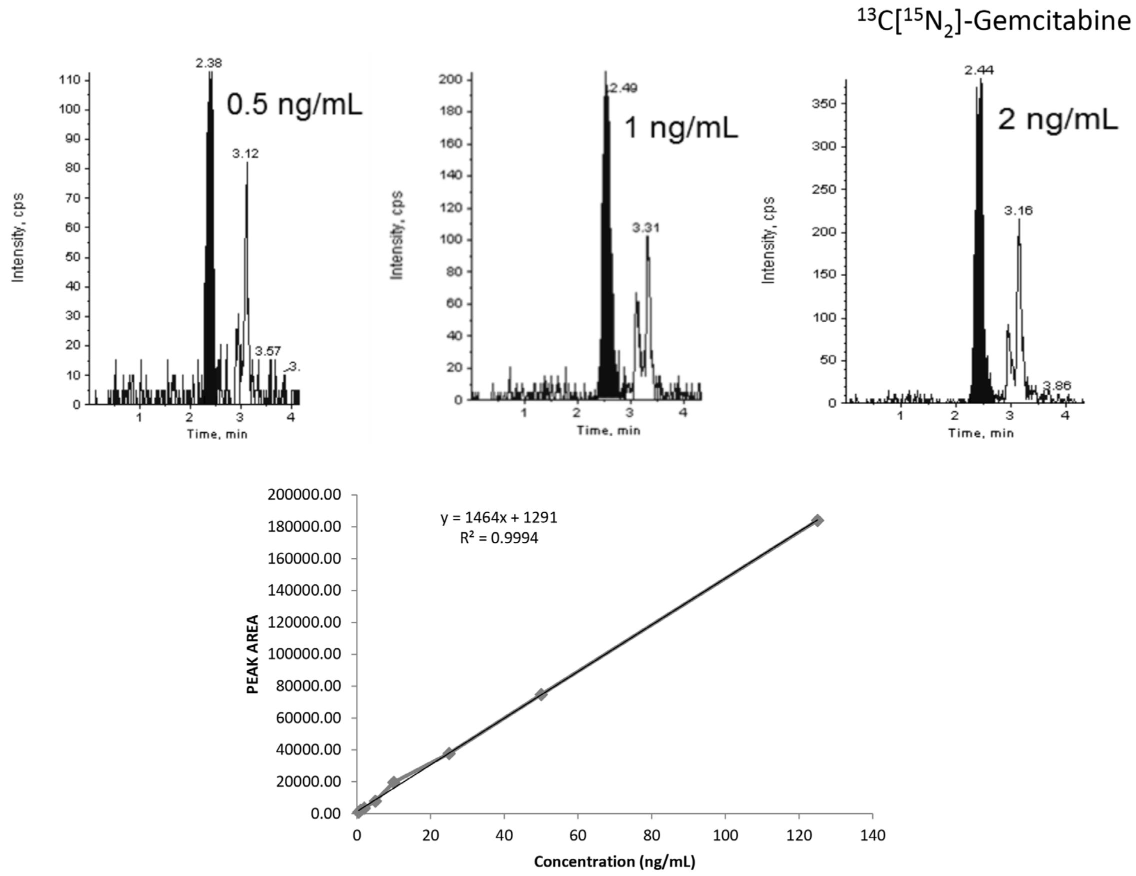

Supplementary Figure S12: A. MVs continue to remove gem from media/microenvironment after their release from cells (inflow assay). The experiment was performed as illustrated, and the presence of both gem and $^{13}C[^{15}N_2]$ gem was analyzed with HPLC-MS/MS. Experiments were repeated twice and results are presented as mean ± SD. **B.** Detection and quantitation with HPLC-MS/MS of $^{13}C[^{15}N_2]$-Gemcitabine. Top Panel: HPLC analysis of compound standards in increasing concentrations (left to right). Bottom Panel: Calibration curve generated from standards.

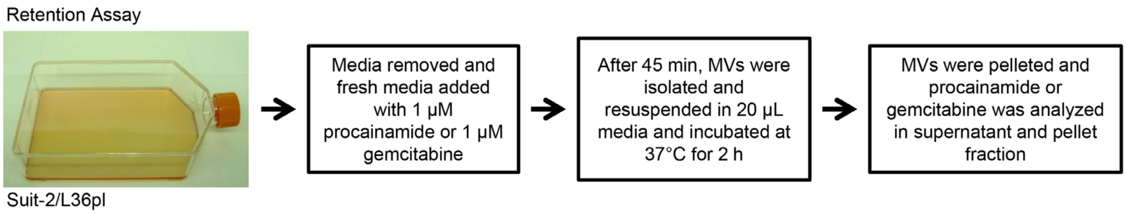

Cells	Incubated with	Fraction analyzed	Procainamide (pg/mg)
Suit-2	Procainamide	supernatant	n/a
	Procainamide	pellet	1292.9 ± 72.8
L36pl	Procainamide	supernatant	n/a
	Procainamide	pellet	1162.1 ± 36.8
			Gemcitabine (pg/mg)
Suit-2	Gemcitabine	supernatant	n/a
	Gemcitabine	pellet	219.0 ± 87.4
L36pl	Gemcitabine	supernatant	n/a
	Gemcitabine	pellet	1292.9 ± 72.8

Supplementary Figure S13: Gem expelled via MVs by Suit-2 is trapped within MVs, while gem expelled via MVs by L36pl cells is released into the microenvironment (retention assay). Suit-2 and L36pl cells were treated with procainamide or gem and released MVs were isolated as outlined in the illustration. The amount of gem or procainamide present in the supernatant or pellet was estimated with UPLC-MS/MS. The experiments were repeated thrice, in duplicate. The mean value is expressed as pg/mg ± SD. MVL= microvesicle lysates; TCL= total cell lysates.

Supplementary Table S1: Histological analysis of tumor volume and necrosis in mice treatment groups

Group	Cells injected	Treatment	Tumor volume (cm^3) ± S.E.	Necrosis (%) ± S.E.
1	Suit-2-luciferase	PBS	10.1 ± 0.5	9.5 ± 1
2	Suit-2-luciferase	Gemcitabine	5.3 ± 0.9	6 ± 1
3	Suit-2-luciferase	AZD6244	0.9 ± 0.7	1 ± 0.5
4	Suit-2-luciferase	Gemcitabine + AZD6244	0	0
5	Suit-2^{T27N}-luciferase	PBS	33.9 ± 1	34 ± 1
6	Suit-2^{T27N}-luciferase	Gemcitabine	9.0 ± 0.8	16 ± 1
